# Effect of postmortem tenderization strategies (pretumbling, blade tenderization, moisture enhancement) on processing yield parameters and eating quality of selected hip and loin muscles from youthful and mature beef carcasses

**DOI:** 10.1093/tas/txab139

**Published:** 2021-08-31

**Authors:** Zeb Pietrasik, Phyllis J Shand

**Affiliations:** Department of Food and Bioproduct Sciences, University of Saskatchewan, Saskatoon, SK S7N 5A8, Canada

**Keywords:** beef, carcass grade, eating quality, processing yield, tenderization strategies

## Abstract

Several muscles from mature beef carcasses have been identified as failing to provide adequate tenderness required for a satisfactory consumer eating experience. Postmortem processing strategies can help improve the tenderness and subsequent eating quality of mature beef muscles. The current study was undertaken to investigate the impact of processing strategies (blade tenderization [BT], pretumbling [PT], and moisture enhancement [ME]), alone and in combination, on processing yield and eating quality-related parameters of selected loin and hip muscles (gluteus medius [GM], longissimus lumborum [LL], semimembranosus [SM], and biceps femoris [BF]) from youthful and mature beef cattle. Results indicate that muscles from mature beef were inherently less tender (*P* < 0.05), but some tenderization procedures produced meat that was similar in tenderness to that of youthful cattle. Of the different tenderization strategies evaluated, BT followed by ME (injection of a salt/phosphate solution) was the most effective strategy for improving (*P* < 0.05) tenderness of tougher muscle cuts such as BF and SM. Moisture enhancement alone improved tenderness (*P* < 0.05) and juiciness (*P* < 0.01) of SM, GM and LL, but with the exception of samples tenderized prior to injection, was not effective (*P* > 0.05) in BF muscles. No additional tenderization of GM and LL samples was observed (*P* > 0.05) by combining PT or BT with ME. Combining PT or BT with ME; however, was effective (*P* < 0.05) to control the increased purge loss observed following ME treatment in SM and LL muscles. Pretumbling as a single treatment was ineffective (*P* > 0.05) in all of the muscles, and only treatments that included BT were sufficient to effect an increase (*P* < 0.05) in tenderness of BF.

## INTRODUCTION

The profitability of the beef cattle industry in Canada is mostly based on the successful production of high-value, youthful carcasses. Nevertheless, the older cull breeding stock represents an important component of the beef production system.

Carcasses from cull cows are generally downgraded in North American grading systems based on assessment of skeletal and lean maturity. Due to inferior eating quality (tenderness, juiciness, flavor) and lack of consistency in tenderness (Stelzleni et al., 2007), beef from mature cull cow carcasses is generally used for further processing and grinding (Holmer et al., 2009; Pivotto et al., 2014; Alvarenga et al., 2021).

Potential opportunities for improving the value of mature carcasses have been explored by Rodas-González et al. (2013), in which cow carcass traits were characterized from mature carcasses graded in the longissimus thoracis muscle according to Canadian standards. The authors found that D1 and D4 carcasses had similar characteristics to youthful carcasses and indicated opportunity to capitalize on the higher marbling content in these grades with adequate intervention strategies for tenderness improvement. Another comprehensive study (Roberts et al., 2018) comparing eating quality of meat from mature beef carcasses with youthful carcasses, showed that while flavor and juiciness of some muscles from graded cow carcasses was equivalent, or even superior, to that of beef from youthful cattle, its tenderness was inferior and failed to provide customer satisfaction. The authors suggested however that some tougher muscles from these mature carcasses may be used in value added products with inclusion of tenderness interventions.

With a mature cow population of 4.5 million (Canfax, 2020), and an annual cull rate of 8–10%, even modest improvements in mature cattle processing to create higher-value beef products could result in substantial industry benefit. Although some cow carcass muscles may never provide a palatable value-added product other than ground beef, the use of one or a combination of further processing technologies could help to mitigate toughness and increase the overall marketability of mature beef.

Injection of ingredients such as sodium tripolyphosphate and sodium chloride that bind water and promote protein solubilization has been well documented to improve the tenderness of less tender beef cuts and aid in meeting the demands of consumers for more consistent, higher quality and convenient meat products (McGee et al., 2003; Hoffman, 2006; Holmer et al., 2009). Blade tenderization can be used on tougher, low-quality muscles to improve ratings for tenderness and detectable connective tissue (Kolle et al., 2004; Pietrasik and Shand, 2010, 2011; Obuz et al., 2014); however, there is concern about the potential food safety risks and consumer awareness associated with the use of invasive enhancement techniques (King et al., 2009; Saha et al., 2019; Yang et al., 2021). Pietrasik and Shand (2005) reported beneficial effects of pretumbling, or massaging, on beef semimembranosus roasts from youthful carcasses and suggested further exploration of the effects of this noninvasive technique on other cuts.

However, although a large body of research exists on the effects of singular use of tenderization techniques, few studies have evaluated potential synergistic effects of the simultaneous use of two or more methods of tenderization for improvement of the palatability of various cuts from mature cattle. The combination of mechanical tenderization with moisture enhancement may provide a useful means of improving eating quality of tougher muscles.

This work was undertaken to investigate the impact of processing strategies (blade tenderization, pretumbling, and moisture enhancement), alone and in combination, on processing yield and eating quality-related parameters of selected loin and hip muscles (gluteus medius, longissimus lumborum, semimembranosus and biceps femoris) from youthful and mature beef cattle.

## MATERIALS AND METHODS

Postmortem samples were obtained from a provincially inspected slaughter facility; therefore, no Institutional Animal Care and Use Committee approval was necessary.

All animals used in this study were humanly slaughtered under the Animal Products Act and under the Meat Inspection (Saskatchewan) Regulations.

### Sample Collection and Treatment

This study was conducted using carcasses graded within the mature cow grades (D1 [excellent muscling with firm white or amber fat] and D2 [medium to excellent muscling, white to yellow fat]; *n* = 12 of each grade) and youthful carcasses of Canada 1 yield grade (Y1 [59% or more of lean, quality grade AA or AAA; *n* = 12).

Twelve youthful beef heifers (average age 17 mo), 12 each of D1 and D2 cows (average age 76 and 88 mo, respectively) were conventionally slaughtered at a provincially inspected abattoir in Saskatchewan. After carcass chilling for 48 h at 4 °C, the selected subprimals including sirloin butt, strip loin, inside round and outside round were removed from both sides of each carcass, labelled to maintain carcass and side identity, vacuum packaged, and shipped to the University of Saskatchewan (Saskatoon, SK) for processing.

All subprimals were aged at 3 °C until 12 d postmortem, then unpackaged and fabricated into gluteus medius (GM), longissimus lumborum (LL), semimembranosus (SM) and biceps femoris (BF), respectively. The muscles were trimmed of all visible fat and connective tissue outside and a small sample was removed from each muscle pair and pooled for subsequent determination of moisture, crude protein, collagen content, and pH. The dorsal end of the GM was separated from the muscle along the existing fat seam and the remainder of the muscle was cut in half perpendicular to the fiber direction to create three muscle locations: dorsal, anterior, posterior. The BF, LL, and SM were each cut into three sections designated as anterior, central, and posterior. Within each muscle pair six treatments were assigned to individual roast sections: untreated (Control), blade tenderization (BT), pretumbling (PT), moisture enhancement (ME), blade tenderization followed by moisture enhancement (BT/ME), or pretumbling followed by moisture enhancement (PT/ME). Roast location within muscle was balanced to ensure that all treatments were assigned twice to each location.

Untreated samples were placed directly into bags, vacuum packaged, and refrigerated for 48 h at 4 °C. The BT and BT/ME roasts were passed once through a tenderizer (Model H #B4590, Jaccard Corporation, Orchard Park, NY). The PT and PT/ME samples were subjected to pretumbling at 8.5 rpm for 30 min at 4–6 °C without vacuum (Vario-Vac tumbler Model #VV1-150, Killvoangen, Germany). Samples designated for ME, BT/ME, and PT/ME treatments were then injected (1.2 bar pressure, 20–24 strokes per min varying with muscle type; Fomaco multineedle injector, Model FGM 20/40, Reiser Ltd, Burlington, ON) to 112% of preprocessing sample weight with brine formulated to deliver 0.4% NaCl and 0.3% sodium tripolyphosphate in the final product. Roasts that did not meet the target injection level after a single pass through the injector (Model No. FGM20/40, Fomaco Reiser, Burlington, ON, Canada) were further treated with a hand-held injector until the target weight was achieved. Processed samples were vacuum packaged and stored at 4 °C. At 48 h postprocessing, each sample was weighed for determination of purge loss ({weight before 48 h storage – weight at 48 h/weight before storage} × 100), then two 2.54 cm-thick steaks were cut from each sample perpendicular to the muscle fiber orientation. The steaks were weighed, vacuum packaged, and blast frozen. One steak from each sample was retained in frozen storage (–30 °C) until required for laboratory evaluation, and the second steak from each was shipped to the Agriculture and Agri-Food Canada Lacombe Research Centre (Lacombe, AB) for evaluation of sensory properties.

### Sample Evaluation

Total moisture (Method No. 950.46 B), crude protein (Method No. 981.10), and total collagen (Method No. 990.26) contents of unprocessed beef were determined in duplicate following AOAC (2000) procedures. The pH values of both unprocessed and processed beef were measured (Accumet 915 pH meter, Fisher Scientific, Nepean, ON) in duplicate using a combination pH electrode on homogenates of 20 g of sample in 80 mL deionized water.

Steaks thawed for analysis were weighed to determine thawing loss ([initial steak weight – thawed steak weight]/initial steak weight × 100). Before measurement of Warner-Bratzler shear force (WBSF) and expressible moisture (EXM), the steaks were thawed at 4 °C for 24 h then cooked on a preheated (210 °C) electric grill (Garland, ED-30B, Condon Barr Food Equipment Ltd, Edmonton, AB). Steaks were placed on the grill, cooked until their internal temperature reached 35 ºC, turned over, and cooked to a final internal temperature of 71 ºC. Steak temperature during cooking was monitored using Type-T copper-constantan thermocouples attached to a scanner (Model 692-8000, Barnant Co., Barrington, IL) and a computer that recorded temperature at 30 s intervals. The cooking time for each steak to reach 71 °C was recorded. Cooked steaks were weighed to determine cooking loss ([weight before cooking – cooked weight]/weight before cooking × 100).

Cooked steaks were allowed to cool at room temperature until their surface temperature reached 25 °C. The shear force of 7–9 core samples (1.27 × 1.27 × 2.54 cm) cut parallel to the fiber direction from each cooked steak was determined. Cores were sheared once perpendicular to the fiber direction using a TMS-Pro texture system (Food Technology Corp., Sterling, VA) fitted with a Warner-Bratzler shear attachment and with crosshead speed set to 200 mm·min^–1^. Maximum peak force recorded during the test was reported was WBSF, while force-deformation curves from the Warner-Bratzler shear device were used to determine shear force of the myofibrillar (SF-M) and connective tissue (SF-C) components of the samples. The initial yield (height of the first peak) corresponded to SF-M and the final yield (height of the second peak) corresponded to SF-C, both of which were measured from the shear curves as previously described (Pietrasik and Shand, 2010); namely, SF-M was measured on the curve as a peak occurring in the first half of the distance the shear blade cut through the meat sample, and SF-C was measured as a peak value within the last 3 mm before the shear blade had completely cut through the sample.

Expressible moisture was determined on grilled steak samples after equilibration to room temperature. Three meat cubes (1.27 × 1.27 × 1.27 cm) were prepared from each steak. Individual, preweighed meat cubes were placed between two layers of Whatman #3 filter paper, compressed perpendicular to the fiber direction to 50% of original height with a 113.4 kg cell at a crosshead speed of 100 mm·min^–1^ on a TMS-Pro texture system, held for 30 s, then released and reweighed before calculating EXM ([initial cube weight – final cube weight]/initial cube weight × 100).

For evaluation of sensory properties, steaks were thawed at 4 °C for 24 h then cooked as previously described for Warner-Bratzler shear force determinations. Cooked steaks were cooled at room temperature for 10 min then prepared into 1.3 cm cubes, placed into glass jars in a circulating water bath and allowed to equilibrate to 68 °C for 7 min prior to serving. The samples for each panelist were presented according to a balanced design assigned by Compusense five software (v 4.6, Compusense, Inc., Guelph, ON, Canada). Panelist training was based on published standards and guidelines (AMSA, 1995) with panelists previously extensively trained for evaluation of meat provided with six additional one hour training sessions for specific attributes. Six samples representing six tenderization treatments within a muscle and grade were evaluated per session, over 144 sessions. Attribute ratings were collected electronically using Compusense five on 8-point descriptive scales for initial tenderness (1= extremely tough, 8 = extremely tender), amount of perceptible connective tissue (1 = abundant, 8 = none detected), juiciness (1 = extremely dry, 8 = extremely juicy), beef flavor intensity (1 = extremely bland, 8=extremely intense), saltiness (1 = extremely intense, 8 = extremely bland), and off-flavor intensity (1 = extremely intense, 8 = extremely bland). Filtered, reverse osmosis water and unsalted soda crackers were provided to cleanse the palate between samples. This study was accepted on ethical grounds (#04-37) by the University of Saskatchewan Behavioral Research Ethics Board.

### Statistical Analysis

Proximate analysis data were analyzed using the PROC MIXED procedure of SAS (SAS Inst. Inc., Cary, NC). The model included fixed effects of quality grade, muscle, and grade by muscle interaction. Carcass nested within grade was specified as random effect. Yield and eating quality-related data were also analyzed with the mixed model procedure. The model included fixed effects of quality grade, muscle, processing treatment, and the two- and three-way interactions. Random effects included carcass nested within grade and the interaction of carcass within grade by muscle. The Satterthwiate’s approximation was used for all denominator degrees of freedom (Littell et al., 1996). Least-squares means were calculated for all main effects or interactions and the means were separated using PDIFF procedure and Tukey test when the respective *F*-tests were significant (*P* ≤ 0.05). The PROC CORR procedure of SAS was used for calculation of Pearson’s correlation coefficients.

## RESULTS AND DISCUSSION

### Characterization of Raw, Unprocessed Beef

Moisture and protein contents did not differ amongst grades (*P* = 0.18 and 0.29, respectively), while collagen content of D2 samples was significantly greater (*P* < 0.01) than that of both Y1 and D1 samples, that did not differ from one another (*P* > 0.05) (Table 1). There was a 12-mo difference in live animal age between samples collected from D1 (average age of 76 mo) and D2 (average age of 88 mo) carcasses and this may have influenced collagen content. In a study conducted with steers between 12 and 20 mo of age, Girard et al. (2011) found that total collagen content increased with slaughter age in GM muscles but was not affected by age at slaughter in ST muscles. Other studies; however, did not find a difference in total collagen content across animal age categories, but observed a significant decrease in soluble collagen content as animal age increased (Schönfeldt and Strydom, 2011; Lucero-Borja et al., 2014; Alvarenga et al., 2021; Roy et al., 2021).

**Table 1.** Mean values of proximate composition (g/100 g) parameters of raw meat samples from each grade and muscle^1^ of beef steaks

		Proximate composition parameter			
Variable	Level	Moisture	Protein	Collagen	pH
Grade	Y1	73.3	21.6	0.69^b^	5.37
	D1	73.8	21.7	0.74^b^	5.41
	D2	73.5	21.5	0.91^a^	5.36
	SEM^2^	0.17	0.09	0.03	0.01
	*P*-value	0.18	0.29	<0.01	0.20
Muscle	LL	72.6^c^	21.8^a^	0.57^c^	5.40
	GM	73.3^b^	21.4^b^	1.02^a^	5.35
	SM	74.3^a^	22.1^a^	0.61^c^	5.37
	BF	73.9^a^	21.0^c^	0.91^b^	5.39
	SEM	0.19	0.10	0.03	0.01
	*P*-value	<0.01	<0.01	<0.01	0.13

^abc^Across the levels of each variable within a column, means without a common superscript differ (*P* ≤ 0.05).

^1^Muscles: BF = biceps femoris; GM = gluteus medius; LL = longissimus lumborum; SM = semimembranosus.

^2^Standard error of the least squares means.

The SM and BF muscles had the highest moisture content (*P* < 0.01), followed by the GM and LL (Table 1). McKeith et al. (1985) reported a similar ranking for moisture content, and other studies also indicated the longissimus to have the lowest moisture content amongst these muscles (Von Seggern et al., 2005; Roberts et al., 2017; Nyquist et al., 2018). Crude protein content increased (*P* < 0.01) from BF to GM, followed by the LL and SM; however, LL and SM did not differ from each other. Collagen content also varied by muscle, with GM having the highest content (*P* < 0.01), followed closely by BF, and then SM and LL. No differences were found between SM and LL (*P* > 0.05). Von Seggern et al. (2005) reported muscles in the same order for collagen content, although the difference between SM and LL was significant. McKeith et al. (1985) also presented muscles by total collagen content and reported BF > GM > SM > LL in youthful steer carcasses. Despite a relatively high collagen content in the GM, Stolowski et al. (2006) and Girard et al. (2011) demonstrated the solubility of collagen in this muscle is also high, bringing it in line with the longissimus, and significantly higher than the SM or ST.

### Processing Yield Parameters

There was no significant interaction between grade and processing treatment for any of the processing yield parameters, indicating that samples from both youthful and mature beef carcasses were similarly affected by various processes. Some yield parameters were affected by both interaction and main effects, and therefore, presentation of these results has been organized by yield parameter.

#### Purge loss.

Amount of purge accumulation during storage was not affected (*P* > 0.05) by different maturity grades and is consistent with results reported by Patten et al. (2008) who observed no differences in WHC between cull cows and youthful carcasses for most muscles examined. Roberts et at. (2017) reported that drip losses from D1 and D2 cow meat were either lower (GM) or not different (BF, SM, LL) than muscles from youthful carcasses. Variable effectiveness of the processing treatments to limit purge loss from whole muscles to 48 h postprocessing led to observation of an interaction between muscle and treatment (*P* = 0.02; Table 2). There were no significant differences (*P* > 0.05) in amount of purge loss among nontreated muscle samples, while purge loss from BF and GM was lower (*P* < 0.05) than from LL and SM in ME treatments. Except for PT/ME samples, purge loss was higher (*P* < 0.05) in treatments where additional moisture had been added to the muscles (ME, BT/ME).

**Table 2.** Effect of muscle^1^ by treatment^2^ interaction on least-squares means of purge loss^3^, pH, and cooking loss of beef steaks

		Processing yield parameter		
Muscle	Treatment	Purge loss, %	pH	Cooking loss, %
LL	Control	1.4^ghijk^	5.58^d^	29.4^ghi^
	BT	1.7^fghi^	5.60^cd^	30.3^defgh^
	PT	1.8^cdefg^	5.61^cd^	29.9^fghi^
	ME	3.2^a^	5.69^ab^	28.8^hi^
	BT/ME	2.4^bc^	5.72^a^	29.5^ghi^
	PT/ME	2.1^cdef^	5.73^a^	28.5^i^
GM	Control	1.1^ijk^	5.44^e^	31.4^cdef^
	BT	1.2^ijk^	5.47^e^	32.5^abc^
	PT	1.2^ijk^	5.48^e^	30.9^cdefg^
	ME	2.2^cde^	5.66^abc^	31.3^cdef^
	BT/ME	1.6^fghi^	5.61^cd^	32.3^abc^
	PT/ME	1.7^efghi^	5.66^abc^	32.2^abc^
SM	Control	1.2^hijk^	5.46^e^	32.0^bcd^
	BT	1.2^hijk^	5.46^e^	33.9^a^
	PT	1.5^ghij^	5.48^e^	32.4^abc^
	ME	2.8^ab^	5.65^bcd^	31.7^bcde^
	BT/ME	2.2^cd^	5.61^cd^	33.5^ab^
	PT/ME	1.8^cdefg^	5.67^abc^	32.0^bcd^
BF	Control	1.0^jk^	5.45^e^	29.4^ghi^
	BT	0.9^k^	5.46^e^	32.4^abc^
	PT	1.0^jk^	5.46^e^	30.2^efghi^
	ME	1.8^defgh^	5.66^abc^	28.5^i^
	BT/ME	1.7^defgh^	5.65^bcd^	32.0^bcd^
	PT/ME	1.3^hijk^	5.67^abc^	29.1^ghi^
SEM^4^		0.14	0.02	0.44
*P*-value		0.02	<0.01	<0.01

^a-k^Within a column, means without a common superscript differ (*P* ≤ 0.05).

^1^Muscles: BF = biceps femoris; GM = gluteus medius; LL = longissimus lumborum; SM = semimembranosus.

^2^Treatments: Control = untreated control; BT = blade tenderization; PT = pretumbling; ME = moisture enhancement by brine injection; BT/ME = blade tenderization followed by moisture enhancement; PT/ME = pretumbling followed by moisture enhancement.

^3^From vacuum packaged samples at 48 h postprocessing.

^4^Standard error of the least squares means.

The ME brine solution had a relatively low ionic strength with a limited capacity to bind the additional moisture. Combining BT or PT with ME significantly reduced purge loss from LL and SM, a finding also reported by Pietrasik and Shand (2005; 2011). Blade tenderization may have served to increase the meat surface area available for interaction between brine and myofibrillar proteins, while the physical manipulation of the muscle tissue during tumbling may have stimulated protein extraction and enhanced the ability of the myofibrillar matrix to bind added water. In each of the muscles, purge loss from BT and PT samples was not different from the untreated Control. This was expected since these treatments did not include additional moisture or the creation of large channels from which moisture could be lost. Some previous studies also reported a lack of effect on purge, or drip loss, following blade tenderization (Tatum et al., 1978; Pietrasik and Shand, 2011; Obuz et al., 2014).

#### pH.

A clear delineation in pH observations existed between samples that were treated with salt/phosphate brine (ME, BT/ME, PT/ME) and those not subject to brine addition (Control, BT, PT). As expected, where phosphate-containing brine was introduced, pH was increased (*P* < 0.01; Table 2). The pH of the LL samples was higher relative to the other muscles, leading to equivalence in pH between mechanically-processed (BT, PT) LL samples and brine-treated BF, GM, and SM samples.

#### Thawing loss.

Each main effect had a small, but significant, influence on weight loss during thawing from previously frozen steaks (Table 3). Steaks from the oldest animals included in the study (D2) had the lowest thawing loss. Only 0.5% unit difference in thaw loss was observed across muscles with LL steaks having significantly (*P* < 0.01) higher loss than SM steaks. While addition of moisture via brine injection (ME, BT/ME, PT/ME) resulted in greater initial purge loss from muscle sections (Table 2), steaks from these muscles had relatively lower losses after thawing. This suggests that after an initial equilibration period, the added moisture tended to remain bound to the tissue.

**Table 3.** Main effects of grade, muscle^1^, and treatment^2^ on least-squares means of thawing loss, cooking time, and expressible moisture^3^ of beef steaks

		Processing yield parameter		
Variable	Level	Thawing loss, %	Cooking time, min	Expressible moisture, %
Grade	Y1	3.5^a^	23.9	15.8^a^
	D1	3.7^a^	23.6	14.8^ab^
	D2	2.6^b^	24.1	14.2^b^
	SEM^4^	0.21	0.60	0.24
	*P*-value	<0.01	0.84	<0.01
Muscle	LL	3.5^a^	22.3^b^	14.8^b^
	GM	3.2^ab^	22.5^b^	14.7^b^
	SM	3.0^b^	23.7^b^	16.2^a^
	BF	3.3^ab^	26.9^a^	14.0^b^
	SEM	0.14	0.43	0.23
	*P*-value	<0.01	<0.01	<0.01
Treatment	Control	3.6^a^	25.8^a^	14.5^bc^
	BT	3.4^ab^	23.9^b^	12.7^d^
	PT	3.6^a^	23.8^b^	13.9^c^
	ME	2.8^c^	23.8^b^	16.5^a^
	BT/ME	3.2^abc^	22.3^c^	15.1^b^
	PT/ME	3.0^bc^	23.6^b^	16.8^a^
	SEM	0.15	0.44	0.23
	*P*-value	<0.01	<0.01	<0.01

^abc^Across the levels of each variable within a column, means without a common superscript differ (*P* ≤ 0.05).

^1^Muscles: BF = biceps femoris; GM = gluteus medius; LL = longissimus lumborum; SM = semimembranosus.

^2^Treatments: Control = untreated control; BT = blade tenderization; PT = pretumbling; ME = moisture enhancement by brine injection; BT/ME = blade tenderization followed by moisture enhancement; PT/ME = pretumbling followed by moisture enhancement.

^3^From cooked meat samples.

^4^Standard error of the least squares means.

Within each main effect, however, the absolute differences between the highest and lowest values were relatively small (0.5–0.9%).

#### Cooking loss.

Cooking loss varied by grade (Y1: 30.0%^b^, D1: 31.7%^a^, D2: 31.3%^ab^, *P* = 0.02; data not shown), although the practical importance of differences of this magnitude would likely be minimal. No significant difference in cooking loss amongst treatments existed within GM, and differences were minimal within the SM (Table 2). In the LL samples only the BT and PT/ME treatments differed from each other and losses from all treatments were not different from the lowest values observed within each of the other muscles. Increased cook loss in longissimus muscles subjected to blade tenderization was reported in other studies (King et al., 2009; Obuz et al., 2014). A distinct delineation between BT and non-BT treatments was observed within the BF muscle (Table 2), where BT and BT/ME resulted in significantly increased cooking loss compared to all other treatments. Pietrasik et al. (2010) reported an increased cooking loss following blade tenderization of the semitendinosus - a muscle also noted for its relatively high connective tissue content. It is possible that the holes created in the muscle tissue by the blade tenderization process were maintained as fluid-exit channels during cooking; a theory also suggested by Davis et al. (1975). Contraction of collagen during cooking may have provided the pressure required to expel moisture during dry-heat cookery (Obuz and Dikeman, 2003).

#### Cooking time.

Biceps femoris samples required an additional 3.2 to 4.6 min (*P* < 0.01) of cooking time to prepare as compared to all other muscles (Table 3). Obuz and Dikeman (2003) also reported longer cooking times for biceps femoris steaks as compared to longissimus steaks. Longer cooking times of steaks from muscles containing high amounts of connective tissue have been reported by others (Girard et al., 2012; Saha et al., 2019). These variations in cooking times may also be related to differences in thermal conductivity, composition, density, specific heat, and muscle fiber orientation with respect to heat transfer (Obuz and Dikeman, 2003). Compared to Control, all processing treatments decreased (*P* < 0.01) the cooking time required to reach an internal sample temperature of 71 °C, with BT/ME requiring the shortest time (Table 3). Similar results have been reported that indicate cooking time is reduced following the application of moisture enhancement (Pietrasik and Shand, 2005; 2010) or blade tenderization (Savell et al., 1977; Pietrasik and Shand, 2011) treatments. These data serve as a reminder to review timed cooking practices for steaks when new tenderization strategies are introduced.

#### Expressible moisture.

Expressible moisture from cooked samples (Table 3) is an indicator of free water content and can be a predictor of expected purge from packaged, precooked, value-added products. As animal age increased, EXM decreased such that cooked steaks from D2 muscles had significantly (*P* < 0.01) lower EXM than did steaks from youthful (Y1) carcasses. Expressible moisture from SM steaks was significantly (*P* < 0.01) greater than from any other muscle. Von Seggern et al. (2005) also reported greater expressible moisture values in the SM compared to both GM and LL, although not significantly different from EXM from BF. Similar to the trend with purge loss, and consistent with the findings of Pietrasik and Shand (2005), steaks from injected muscles had significantly higher EXM than steaks made from uninjected meat, indicating that brine addition resulted in more free water in the samples. The exception was BT/ME, in which the physical treatment countered the influence of injection to produce samples with EXM not different from the control. Where BT was applied as a sole processing treatment, EXM was significantly lower than from Control, likely due to the greater losses from these samples already incurred during cooking. With its lesser degree of physical disruption, there was no difference from Control in EXM from PT samples; a result also reported by Pietrasik and Shand (2005).

### Eating Quality-Related Parameters

#### WBSF and sensory tenderness.

Within each grade, there was no difference in WBSF or sensory tenderness scores between GM and LL muscles (Table 4). These two muscles had the lowest shear values and highest tenderness scores at every grade level, and had the least amount of sensory perceptible connective tissue (BF: 3.8^c^, SM: 4.9^b^, GM: 5.7^a^, LL: 6.0^a^, where 1 = abundant, 8 = none detected, *P* < 0.01; data not shown). The same relationship between connective tissue sensory scores and these two muscles has been reported by Wheeler et al. (2000) and Rhee et al. (2004). Roberts et al. (2018) also reported similarly; however, an additional significant difference was reported between GM and LL. Greater amount of perceived connective tissue in round muscles observed by the trained panelists in our study is in agreement with previous reports linking an increased level of connective tissue to lower sensory tenderness scores (Nyquist et al., 2018) or higher shear force values (Alvarenga et al., 2021).

**Table 4.** Effect of grade by muscle^1^ interaction on least-squares means of Warner-Bratzler shear force (WBSF) values, and sensory scores for tenderness^2^ and juiciness^3^ of beef steaks

		Eating quality-related parameters		
Grade	Muscle	WBSF, N	Tenderness	Juiciness
Y1	LL	39.17^f^	6.3^a^	4.3^de^
	GM	47.67^ef^	6.0^a^	4.3^de^
	SM	60.16^cd^	5.2^b^	4.1^e^
	BF	68.42^c^	4.7^b^	5.1^a^
D1	LL	52.37^de^	5.0^b^	4.4^bcde^
	GM	58.53^d^	5.0^b^	4.2^de^
	SM	79.25^b^	3.9^c^	4.1^e^
	BF	101.42^a^	3.2^d^	4.8^abc^
D2	LL	51.50^de^	4.8^b^	4.5^bcd^
	GM	56.57^d^	5.1^b^	4.4^cde^
	SM	83.72^b^	3.9^c^	4.2^de^
	BF	109.20^a^	3.1^d^	4.8^ab^
SEM^4^		2.79	0.15	0.10
*P*-value		<0.01	0.04	0.02

^a-f^Within a column, means without a common superscript differ (*P* ≤ 0.05).

^1^ Muscles: BF = biceps femoris; GM = gluteus medius; LL = longissimus lumborum; SM = semimembranosus.

^2^1 = extremely tough, 8 = extremely tender.

^3^1 = extremely dry, 8 = extremely juicy.

^4^Standard error of the least squares means.

Furthermore, GM and LL samples from D1 and D2 carcasses were equivalent in tenderness to BF and SM samples from youthful carcasses. Y1 samples from each muscle were always more tender (lower shear force, higher tenderness score) than D1 samples, with no further increases in toughness observed for the D2 samples. WBSF values for GM, LL, SM, and BF were 23%, 34%, 32%, and 48% higher, respectively for D1 versus Y1 muscles. This finding is in agreement with previous research showing higher shear force and/or lower tenderness ratings in meat from beef cull cows (Stelzleni et al., 2007; Schönfeldt and Strydom, 2011; Lucero-Borja et al., 2014; Roberts et al., 2018; Alvarenga et al., 2021).

Roberts et al. (2018) compared eating quality and shear force of eleven muscles from mature beef carcasses (D1-D4) with meat from youthful carcasses. They found that, with the exception of equivalency of WBSF values between Y1 and D1 for longissimus (LL), psoas major (PM) and infraspinatus (IF) and sensory tenderness of PM, all other muscles of the D1 carcass were less tender (higher WBSF, lower tenderness scores) than muscles from youthful carcasses. For D2 carcasses, all respective muscles had a higher shear force and corresponding lower scores for initial and overall tenderness than meat from youthful cattle. Alvarenga et al. (2021) suggested that tenderness/toughness may not be directly related to the age or maturity of cattle, but rather linked to muscle function within carcass which influence the amount and distribution of connective tissue across different muscles of the carcass. Substantiating this, Roy et al. (2021) found no difference in mean peak WBSF values between the market age categories (11–73 mo of age) for the GM muscle but reported significant increase in WBSF values with advancing age for semitendinosus muscle.

An interaction between muscle and treatment was observed for both instrumental and sensory measures of tenderness, with the outcome of processing treatments appearing to be somewhat dependent on the relative contribution of the myofibrillar and connective tissue components of each muscle. The GM and LL muscles were not different from each other in terms of WBSF and tenderness scores, and the influence of the various treatments on WBSF was the same for both muscles (Table 5). In both the GM and LL, only treatments that included salt/phosphate injection (ME, ME/BT, PT/ME) resulted in a significant change in WBSF. Each of these treatments reduced shear values, although where ME was applied, there was no additional benefit to combining injection with BT or PT. A similar trend across treatments in initial tenderness scores of the GM and LL muscles was observed, with the exception that BT increased the tenderness score for GM relative to its control, and combining BT with ME increased LL tenderness relative the tenderness score when ME was applied as a sole treatment to LL. While blade tenderization has been shown to be effective for tougher cuts (Jeremiah et al., 1999; Obuz et al., 2014) some previous studies reported that mechanical tenderization offered no advantage when used on meat that was already of acceptable tenderness (Tatum et al., 1978). WBSF data from the present study supported this conclusion; however, sensory tenderness scores indicated otherwise, showing tenderness improvements with BT of LL and GM. Supplementary consumer testing could provide useful insight about the impact of the processing treatments on product acceptability.

**Table 5.** Effect of muscle^1^ by treatment^2^ interaction on least-squares means of Warner-Bratzler shear force (WBSF) values, shear force of the myofibrillar tissue component (SF-M), shear force of the connective tissue component (SF-C), and sensory scores for tenderness^3^, amount of connective tissue^4^, and juiciness^5^ of beef steaks

		Eating quality-related parameter					
Muscle	Treatment	WBSF, N	SF-M, N	SF-C, N	Tenderness^3^	Connective tissue^4^	Juiciness^5^
LL	Control	59.26^ghi^	58.11^def^	41.36^ijkl^	4.7^def^	5.7^cdef^	4.2^efg^
	BT	54.22^ghijk^	53.14^fgh^	38.18^klm^	5.1^cd^	5.9^bcd^	3.9^fghi^
	PT	49.03^ijkl^	48.23^ghi^	36.74^klm^	5.2^cd^	6.0^abc^	4.0^fgh^
	ME	44.25^kl^	42.42^ij^	34.38^lm^	5.3^bc^	6.1^abc^	4.8^cd^
	BT/ME	39.50^l^	38.28^j^	30.04^m^	6.0^a^	6.4^a^	4.7^cd^
	PT/ME	39.81^l^	37.59^j^	32.48^lm^	5.8^ab^	6.2^ab^	4.9^c^
GM	Control	64.87^efg^	63.23^cde^	51.09^ghi^	4.7^def^	5.4^ef^	3.8^ghij^
	BT	55.48^ghij^	54.38^fg^	45.38^hijk^	5.3^c^	5.9^bcd^	3.5^ij^
	PT	58.11^fgi^	55.30^efg^	50.80^ghij^	5.1^cde^	5.6^def^	3.9f^ghij^
	ME	52.29^hijk^	48.92^ghi^	46.23^hijk^	5.6^abc^	5.8^bcde^	5.0^bc^
	BT/ME	45.74^jkl^	44.11^ij^	40.28^jklm^	6.0^a^	6.1^abc^	4.7^cd^
	PT/ME	49.05^ijkl^	45.56^hij^	45.17^hijk^	5.8^abc^	5.7^cdef^	4.8^cd^
SM	Control	89.19^bc^	82.81^a^	76.95^cd^	3.7^ij^	4.4^ij^	3.9^fghij^
	BT	73.01^de^	67.75^bc^	90.83^fg^	4.5^f^	4.9^gh^	3.5^j^
	PT	80.40^cd^	75.41^ab^	70.99^def^	3.9^ghi^	4.4^i^	3.7^hij^
	ME	71.33^def^	63.16^cde^	65.44^ef^	4.4^fg^	4.7^hi^	4.7^cd^
	BT/ME	60.72^fgh^	55.08^efg^	54.62^gh^	5.2^cd^	5.3^fg^	4.2^ef^
	PT/ME	71.60^de^	63.03^cde^	67.70^def^	4.6^ef^	4.9^h^	4.8^cd^
BF	Control	103.76^a^	81.76^a^	100.35^a^	3.1^k^	3.4^m^	4.8^cd^
	BT	87.47^bc^	75.56^ab^	83.90^bc^	3.9^hi^	4.0^jk^	4.1^efg^
	PT	100.20^a^	79.47^a^	98.09^a^	3.3^jk^	3.5^lm^	4.5^de^
	ME	96.95^ab^	68.64^bc^	95.39^a^	3.7^ij^	3.8^klm^	5.5^a^
	BT/ME	74.54^de^	58.32^def^	71.73^de^	4.4^fgh^	4.4^ij^	5.0^bc^
	PT/ME	95.14^ab^	65.79^cd^	94.33^ab^	3.7^ij^	3.9^kl^	5.3^ab^
SEM^6^		2.73	2.10	2.75	0.13	0.11	0.10
*P*-value		<0.01	0.01	<0.01	0.04	<0.01	<0.01

^a-m^Within a column, means without a common superscript differ (*P* ≤ 0.05).

^1^Muscles: BF = biceps femoris; GM = gluteus medius; LL = longissimus lumborum; SM = semimembranosus.

^2^Treatments: Control = untreated control; BT = blade tenderization; PT = pretumbling; ME = moisture enhancement by brine injection; BT/ME = blade tenderization followed by moisture enhancement; PT/ME = pretumbling followed by moisture enhancement.

^3^1 = extremely tough, 8 = extremely tender.

^4^1 = abundant, 8 = none detected.

^5^1 = extremely dry, 8 = extremely juicy.

^6^Standard error of the least squares means.

Only BT and BT/ME treatments reduced WBSF of the BF relative to its control, while sensory tenderness scores were improved with each of the treatments except PT (Table 5). Regardless, even the most substantial effect was only able to reduce WBSF and increase sensory tenderness scores of BF to the level of GM and LL control. Tenderization of the BF muscle is particularly challenging due to the large contribution of the connective tissue fraction to overall WBSF (Table 6). Only BT and BT/ME were effective to reduce SF-C. The other treatments were ineffective (PT) or had a primary influence not on SF-C but on SF-M (ME, PT/ME), and SF-M is not an important driver of WBSF in BF (Pietrasik and Shand, 2010).

**Table 6.** Pearson correlation coefficients (*P* <0.01) between Warner-Bratzler shear force (WBSF) and each of shear force of the myofibrillar tissue component (SF-M), and shear force of the connective tissue component (SF-C) within each muscle^1^

	Correlation coefficient	
Muscle	SF-M	SF-C
LL	0.99	0.82
GM	0.97	0.90
SM	0.92	0.91
BF	0.85	0.98

^1^Muscles: BF = biceps femoris; GM = gluteus medius; LL = longissimus lumborum; SM = semimembranosus

The resistance of biceps femoris muscle to tenderization treatments seen in the present study is consistent with other studies. Kolle et al. (2004) suggested the relatively large amount of collagen in the BF may contribute to the lack of tenderization effect. Tenderization treatments for BF must be focused on affecting connective tissue with the use of disruptive physical techniques or chemical/enzymatic amendment, although even the most aggressive processes may be insufficient (Pietrasik and Shand, 2010) to provide adequate consumer satisfaction.

In the SM, each of the treatments, except PT, reduced WBSF relative to SM control. Combining BT or PT with ME did not result in further tenderization beyond the shear force reduction observed with ME alone (Table 5). SF-M and SF-C have similarly substantial relationships to WBSF (Table 6), and both BT and ME alone, and in combination, were effective to reduce both SF-M and SF-C, with PT/ME also effective on SF-M.

Moisture enhancement with brine containing sodium chloride and phosphates was more effective in muscles in which hardening of myofibrillar proteins was considered to be the main component of WBSF. Its effect was minor in muscles where the connective tissue component provided the main contribution towards maximum WBSF. Thus, it appears that moisture enhancement targets mainly myofibrillar proteins without affecting connective tissue, and thus, injection with salt/phosphate solution may be ineffective for improvement the palatability characteristics of muscles high in connective tissue. The cutting action of blade tenderization is a more effective means of disrupting muscle fibers and connective tissue to reduce shear force values in most cuts, including those with a substantial connective tissue component. While SM and BF are adjacent to each other in the round, properties of SM seemed to be intermediate to that of GM/LL and BF when cooked quickly as steaks. It responded well to ME (possibly due to its low fat content), but also to BT due to its connective tissue amount. It would be interesting to see if this same relationship holds with long, slow high moisture cooking.

#### Amount of perceptible connective tissue.

Across all treatments, including the control, less connective tissue was perceived in Y1 samples than in samples from D1 and D2 carcasses (Table 7). This finding is consistent with Roberts et al. (2018), who associated the increased panel scores for perceptible connective tissue in D graded carcasses with an increase in collagen crosslinking in mature cattle. The Y1 samples were perceived to be relatively low in connective tissue even without treatment (average score of 5.7–6.3, indicating slight to trace amounts of connective tissue). Only the most invasive processing combination (BT/ME) was sufficient to effect any significant further reduction in the amount of perceptible connective tissue. This same treatment was also effective in reducing perceived connective tissue content of D1 samples to the level of the Y1 control. Relative to their own control, perception of connective tissue in D1 samples was reduced by BT, BT/ME, and PT/ME treatments. Not surprisingly, given its significantly greater collagen content (Table 1), reduction by processing treatment of perceived connective tissue content of D2 samples to make them comparable to Y1 were not observed, and only the BT/ME process was sufficient to effect a reduction relative to the D2 control.

**Table 7.** Effect of grade by treatment^1^interaction on least-squares means of sensory scores for amount of connective tissue^2 (*P*^ =0.02, SEM^3=0.12^) of beef steaks

	Grade		
Treatment	Y1	D1	D2
Control	5.7^bc^	4.3^g^	4.2^g^
BT	6.0^ab^	4.9^def^	4.6^fg^
PT	5.9^ab^	4.5^fg^	4.2^g^
ME	6.0^ab^	4.7^efg^	4.6^fg^
BT/ME	6.3^a^	5.3^cd^	5.1^de^
PT/ME	6.0^ab^	4.9^def^	4.7^efg^

^a-g^Means without a common superscript differ (*P* ≤ 0.05)

^1^Treatments: Control = untreated control; BT = blade tenderization; PT = pretumbling; ME = moisture enhancement by brine injection; BT/ME = blade tenderization followed by moisture enhancement; PT/ME = pretumbling followed by moisture enhancement

^2^1 = abundant, 8 = none detected

^3^Standard error of the least squares means

The interaction between muscle and treatment indicated a variable impact of processing on perceived connective tissue content (Table 5). As sole treatments, PT and ME were ineffective to reduce connective tissue perception in any of the muscles relative to their controls, while BT reduced connective tissue perception in BF, GM, and SM samples. The PT/ME combination treatment was effective in BF, LL, and SM, and BT/ME had a positive impact on all muscles.

#### Juiciness.

A small, but statistically significant (*P* = 0.02) interaction effect between grade and muscle was observed for juiciness scores, primarily due to the distinction between BF and each of the other muscles within the Y1 grade category (Table 4). In agreement with Roberts et al. (2018), the BF samples from youthful carcasses were juicier compared to all other muscles sampled from Y1 carcasses. Within D1 and D2 grades, the juiciness of BF and LL was not different. As animal age increased, the relative difference amongst the muscles decreased; however, the SM was always the least juicy. Juiciness scores of the muscles examined in this study were not affected (*P* > 0.05) by maturity grades. This is consistent with results reported by Roberts et al. (2018) who observed no differences in juiciness scores between cull cows and youthful carcasses for most muscles examined. They found that out of eleven muscles examined only Rectus femoris, Longissimus lumborum, Psoas major and Teres major in the D-graded carcasses were scored as more juicy compared to youthful carcasses. Increased juiciness with higher chronological age has also been observed in meat from cull cows and bulls (Dransfield et al., 2003).

An interaction between muscle and processing treatment also influenced juiciness scores (*P* < 0.01, Table 5). Across all muscles, the treatments that included ME were juiciest; although BF and SM control samples were juicy enough so as to be indistinguishable from their counterpart BT/ME samples. In the GM, LL, and SM muscles, none of the ME-related treatments was sufficient to increase sample juiciness above that of BF control samples. The beneficial effect of moisture enhancement on sensory juiciness was consistent with our previous findings (Pietrasik and Shand, 2010; 2011) and other published reports in which phosphate/sodium chloride improved the juiciness of injected beef (Vote et al., 2000; Robbins et al., 2002; McGee et al., 2003; Hoffman, 2006; Holmer et al., 2009). In general, the noninvasive PT treatment did not affect juiciness as compared to Control, while BT lowered juiciness in the relatively moist BF muscle.

#### Beef flavor, saltiness, off-flavor.

Similar to a previous report (Roberts et al., 2018), the panel scores indicate that the muscles from the mature carcasses had similar flavor intensities to those obtained from youthful carcasses.

Flavor-related sensory parameters were affected only by the main effect of processing treatment (Table 8). Although statistically significant (*P* < 0.01), the 0.2 scale unit difference indicates that beef flavor was relatively unchanged by the various processing treatments. As expected, where salt/phosphate injection was included, samples were scored as more intensely salty (*P* < 0.01). Treatments that were noninvasive (Control, PT) had slightly, but significantly, more intense off-flavor than treatments that included ME. Although the absolute difference was less than 0.5 scale units, this indicates that inclusion of injected ingredients may mask inherent meat flavors.

**Table 8.** Main effect of treatment^1^ on least-squares means of sensory scores for beef flavor intensity^2^, saltiness^3^, and off-flavor intensity^3^ of beef steaks

	Flavor parameter		
Treatment	Beef flavor intensity	Saltiness	Off-flavor intensity
Control	3.9^ab^	7.4^a^	6.4^c^
BT	4.0^a^	7.4^a^	6.6^bc^
PT	3.8^b^	7.4^a^	6.4^c^
ME	3.8^b^	6.2^b^	6.7^a^
BT/ME	3.9^a^	6.2^b^	6.8^a^
PT/ME	3.8^b^	6.1^b^	6.7^ab^
SEM^4^	0.04	0.02	0.03
*P*-value	<0.01	<0.01	<0.01

^a-c^Within a column, means without a common superscript differ (*P* ≤ 0.05)

^1^Treatments: Control = untreated control; BT = blade tenderization; PT = pretumbling; ME = moisture enhancement by brine injection; BT/ME = blade tenderization followed by moisture enhancement; PT/ME = pretumbling followed by moisture enhancement

^2^1 = extremely bland, 8 = extremely intense

^3^1 = extremely intense, 8 = extremely bland

^4^Standard error of the least squares means

## CONCLUSIONS

Carcass grade affected proximate composition, particularly total collagen content, with the most mature carcasses (D2) having significantly more collagen than youthful (Y1) and D1 carcasses. Although the BF and SM muscles were always less tender than GM and LL, increased carcass maturity substantially increased WBSF in the former, but not in the later, with no significant difference in shear observed between D1 and D2 grades in any of the muscles. Beef from mature carcasses would require treatment in order to reduce sensory perceptible connective tissue, although even the most rigorous tenderness enhancement technique may not be sufficient to realize equivalence with youthful beef.

The tenderization response to processing treatments was largely muscle-dependent. Blade tenderization followed by moisture enhancement with salt/phosphate brine injection was effective across all muscles, and ME as a single treatment effectively reduced WBSF of muscles with lower perceptible connective tissue. PT was effective in none of the muscles. Although no additional gains in tenderness of LL and GM were realized when ME was combined with BT or PT, these additional treatments were effective to control the higher purge loss observed where brine was added to the muscles. In the BF, with its greater perceptible connective tissue, only tenderization treatments that included BT were sufficient to reduce WBSF. BT alone significantly reduced shear values, with further reduction observed when BT was combined with ME. Sensory tenderness scores of BF, and both sensory scores and WBSF of SM, were significantly improved with the application of any of the processing treatments except PT. Blade tenderization treatments significantly increased cooking loss from BF, but this was also the juiciest muscle regardless of treatment. The tenderization treatments had a minimal impact on flavor attributes. Regardless of carcass grade, muscle-specific intervention strategies are recommended to achieve maximum value from a beef carcass while maintaining processing efficiency by applying only the most appropriate tenderization techniques.
